# Simulated dive in rats lead to acute changes in cerebral blood flow on MRI, but no cerebral injuries to grey or white matter

**DOI:** 10.1007/s00421-012-2565-8

**Published:** 2012-12-12

**Authors:** Marianne B. Havnes, Marius Widerøe, Marte Thuen, Sverre H. Torp, Alf O. Brubakk, Andreas Møllerløkken

**Affiliations:** 1 Department of Circulation and Medical Imaging, Norwegian University of Science and Technology, 7489 Trondheim, Norway; 2 Department of Laboratory Medicine, Children’s and Women’s Health, Norwegian University of Science and Technology, 7489 Trondheim, Norway

**Keywords:** Diving, Cerebral circulation, MRI, Histology

## Abstract

In this study, the effect of a simulated dive on rat brain was investigated using several magnetic resonance imaging (MRI)-methods and immunohistochemistry. Rats were randomly assigned to a dive- or a control group. The dive group was exposed to a simulated air dive to 600 kPa for 45 min. Pulmonary artery was monitored for vascular gas bubbles by ultrasound. MRI was performed 1 h after decompression and at one and 2 weeks after the dive with a different combination of MRI sequences at each time point. Two weeks after decompression, rats were sacrificed and brains were prepared for histology. Dived rats had a different time-curve for the dynamic contrast-enhanced MRI signal than controls with higher relative signal intensity, a tendency towards longer time to peak and a larger area under the curve for the whole brain on the acute MRI scan. On MRI, 1 and 2 weeks after dive, T_2_-maps showed no signal abnormalities or morphological changes. However, region of interest based measurements of T_2_ showed higher T_2_ in the brain stem among decompressed animals than controls after one and 2 weeks. Microscopical examination including immunohistochemistry did not reveal apparent structural or cellular injuries in any part of the rat brains. These observations indicate that severe decompression does not seem to cause any structural or cellular injury to the brain tissue of the rat, but may cause circulatory changes in the brain perfusion in the acute phase.

## Introduction

Saturation diving is widely used for maintenance and inspection of off-shore subsea systems. Exposure to hyperbaric environments is associated with risk of developing decompression sickness (DCS), arterial gas embolism, neurological symptoms and pulmonary dysfunction (Francis et al. 1990; Mollerlokken et al. 2011; Neuman 2003). Amongst the self-reported symptoms following decompression are conspicuous fatigue, visual disturbances, dizziness, nausea and changes in skin sensitivity (Brubakk et al. 1994). The latter symptoms may be related to the effects of hyperbaria on the central nervous system (CNS).

The long-term health effects of diving have been a matter for discussion for many years, and several studies have been performed to examine the neurological effects of diving (Erdem et al. 2009; Irgens et al. 2007). Saturation divers have reported problems with concentration and memory more frequently than control subjects (Ross et al. 2007; Todnem et al. 1990). In recreational diving accidents, neurological symptoms such as numbness, paraesthesia, dizziness and coordination deficiencies are among the most often reported manifestations of DCS (Vann et al. 2011; Newton et al. 2007).

DCS is a clinical diagnosis associated with a number of different signs and symptoms (Vann et al. 2011). However, in serious DCS neurological symptoms dominate (Moon and Gorman 2003). When neurological damage occurs in divers, the suspected primary cause is vascular gas bubbles. Vascular gas bubbles can enter the arterial circulation by a number of methods, but most commonly due to a right-to-left shunt in the heart, or after barotrauma (Warren et al. 1988). Vascular bubbles can have mechanical, embolic and biochemical effects (Mollerlokken et al. 2011). Entrapment of these bubbles may lead to cellular injury, cerebral edema and increased permeability of the blood–brain barrier (BBB) (Hjelde et al. 2002; Kaakkola et al. 1982). Acute effects can be caused by extravascular bubbles producing pain, or vascular bubbles obstructing and causing stroke-like symptoms (Vann et al. 2011). High intensity signals in the white matter on T_2_ weighted magnetic resonance images (MRI) have been seen in divers after dive injuries (Cordes et al. 2000; Hutzelmann et al. 2000), while interestingly, cerebral abnormalities observed in case studies seem to appear at different times-points and are of varying permanence (Jallul et al. 2007). Abnormal electroencephalography (EEG) results have been noted in the temporal or frontal regions after recompression treatment in divers with cerebral DCS (Gronning et al. 2005). The presence of vascular bubbles does not mean that DCS will occur, but the absence of bubbles is considered to be a good indicator of decompression safety (Sawatzky 1991).

Bubbles can also lead to delayed symptom onset in relation to the vascular system. Endothelial dysfunction has been observed after diving in experiments with animals (Nossum et al. 2002) and humans (Brubakk et al. 2005), and is believed to be caused by vascular bubbles. Inflammatory responses have also been investigated in relation to bubble formation and decompression (Bigley et al. 2008). To what extent vascular bubbles and decompression per se affect long-term health, if at all, is still controversial.

In the present study, the effect of a simulated dive on the rat brain was investigated using several MRI-methods, at 1 h, 1 and 2 weeks after decompression. The rat brains also underwent light microscopical examination including immunohistochemistry.

## Methods

Fourteen Sprague-Dawley (Taconic, Denmark) rats weighing 310.75 ± 17.42 g were used in the study. All experimental procedures and the care of experimental animals conformed to the European Convention for the protection of vertebrate animals used for experimental and other scientific purposes, and the protocol was approved by the Norwegian Council for Animal Research.

### Dive protocol

Following 1 week of acclimatization, the rats were randomly assigned to one of two groups, diving (*n* = 9) or control (*n* = 5). The rats in the dive group were exposed to a simulated air dive in a 20 L hyperbaric chamber with a continuous air supply. They were compressed at a rate of 200 kPa/min to a pressure of 600 kPa, maintained at that pressure for 45 min and then decompressed to the surface (100 kPa) at a rate of 50 kPa/min. The control group was kept in their housing facilities at 100 kPa (normal ambient pressure), breathing air.

### Ultrasound

Immediately after surfacing, the rats were anaesthetized (1 % isoflurane mixed with medical air), and the pulmonary artery was monitored for vascular gas bubbles for 30 min by transthoracic echocardiography using a 35 MHz probe (Vevo770, Visual Sonics, Toronto, ON, Canada). Bubbles were identified on the monitor as bright spots in the pulmonary artery and verified with Doppler. The amount of bubbles in the pulmonary artery was graded on a 0–5 scale according to a previously described method (Eftedal and Brubakk 1997), where bubble grade 0 = no bubbles, 1 = occasional bubbles, 2 = at least one bubble/4th heart cycle, 3 = at least one bubble/heart cycle, 4 = continuous bubbling and 5 = massive bubbling, also described as “white-out” as individual bubbles cannot be seen. The animals were observed for signs of neurological decompression sickness, such as walking difficulties or paresis, before and after the MRI.

### Magnetic resonance imaging

MRI was performed 1 h after decompression (acute MRI) and at 1 and 2 weeks after the dive, with a different combination of MRI sequences at each time point. All MRI was performed using a 7 Tesla magnet (Biospec 70/20 AS, Bruker Biospin MRI, Ettlingen, Germany) with water-cooled (BGA-12, 400 mT/m) gradients. A volume resonator was used for RF transmission, and an actively decoupled rat head surface coil was used for RF reception (Bruker Biospin MRI). During scanning, the anaesthetized (Isoflurane 4 % induction and 2 % maintenance in 30 % O_2_, 70 % N_2_) rats lay prone in a dedicated water heated rat bed. The head of every animal was fixed in the same position with inbuilt earplugs, tooth bar and nose-mask, to assure the same placement within the magnet from scan to scan.

Acute MRI was performed after the ultrasound, within 1 h after the dive. Before the MRI acquisition, the rats were anaesthetized and a 25 Gauge neoflon was inserted in the tail vein. After a gradient echo, fast low angle shot (FLASH) pilot scan (acquisition time 1 min), a series of T_2_-weighted images were obtained to visualise anatomical changes and oedema using a turbo spin echo (rapid acquisition with relaxation enhancement, RARE) sequence with RARE-factor = 4, effective echo time (TE) = 25/50/75 ms, repetition time (TR) = 4,000 ms, 3 averages, acquisition time 7 min 12 s. Diffusion weighted images were obtained using an echo planar imaging sequence with 3 directions and 6 b-values (100/200/400/600/800/1,000 ms), TE = 53.47 ms, TR = 3,000 ms, 6 averages, acquisition time 8 min 24 s. For all of these scans, 17 coronal slices were acquired.

To visualise acute changes in brain perfusion and possible disruption of the BBB, dynamic contrast enhancement (DCE) studies were performed: at first, a T_1_-map was acquired using a RARE sequence with RARE-factor = 4, TE = 7.1 ms, TR = 341/562/845/1,243/1,913/5,000 ms, 1 average and acquisition time 7 min 55 s. This was followed by a pre-contrast T_1_-weighted image (RARE): TE = 7.0 ms, TR = 350 ms, 8 averages and acquisition time 1 min 41 s. Acquisition matrix (MTX) was 256 × 144 zero-filled to 256 × 192 giving an isotropic in-plane resolution of 156 × 156 μm^2^. Thereafter, a time-series of T_1_-weighted images was acquired (RARE-factor = 4, TE = 7.0, TR = 300 ms, MTX 128 × 72, zero-filled to 128 × 96, 1 average). With each image taking 5.4 s to acquire, 200 images were acquired over 18 min. After 60 s of acquisition, a dose of 0.3 mmol/kg 0.25 M gadolinium-based contrast agent (Omniscan, GE Healthcare, United Kingdom) (total volume ~0.36 ml) was injected intravenously through the neoflon over a period of 5 s. Finally, a post-contrast T_1_-weighted image was acquired with the same parameters as the pre-contrast T_1_-weighted scan. For all acquisitions, the field of view (FOV) was 40 × 30 mm and 7 slices á 1 mm was acquired.

The next day, animals were injected with a single dose of 40 mg MnCl_2_ (# 7773-01-5, Sigma-Aldrich Inc., St. Louis, USA) per kg bodyweight (~318 μmol Mn^2+^/kg) at a concentration of 100 mM intra-peritoneally to serve as MRI contrast for detecting subsequent inflammation and gliosis 1 week after (Widerøe et al. 2009).

On the follow-up, MRI made seven and 14 days after the dive and T_2_-weighted images were acquired with the same parameters as described for the acute MRI to study anatomical changes and possible edema associated with tissue pathology. In addition, to evaluate manganese-uptake, a 3D data set was obtained on day seven using a T_1_-weighted gradient echo FLASH sequence with flip-angle = 30°, TR = 12 ms, TE = 3.25 ms. FOV = 30 × 35 × 20 mm and MTX was 192 × 168 × 96 zero-filled to 192 × 224 × 128 and the interpolated resolution was 156 μm isotropic. Images were averaged 16 times and acquisition time was 52 min with 16 averages. The transmit field of the volume-coil was considered homogeneous within the FOV, while the spatially inhomogeneous sensitivity of the surface coil used in the 3D T_1_-weighted FLASH acquisition was corrected for using two additional scans in coupled and single coil operation: 3D T_1_-weighted FLASH sequences with the same FOV and contrast parameters as described above but with matrix size 32 × 32 × 32. Acquisition time was two min for each scan. Correction of the high resolution 3D data set was performed using in-house developed software (MATLAB ver. R2010a), and is described in detail elsewhere (Widerøe et al.  2009).

On both 7 and 14 days after the decompression, diffusion tensor imaging (DTI) was performed to evaluate specific white matter injury and to look for changes in white matter. The DTI was acquired with an Echo planar imaging sequence using 30 directions and *b* = 1,000 ms, 5 images with *b* = 0 ms: FOV = 40 × 40 mm, MTX = 172 × 172 giving a resolution of 233 × 233 μm^2^. On day 7, TE = 37.5, TR = 3,000 ms, 17 slices á 1 mm were acquired with two averages giving an acquisition time of 14 min. On day 14 TE = 37.5, TR = 5,000 ms, 33 slices at 0.5 mm were acquired with six averages giving an acquisition time of 1 h 10 min.

### MR image analysis

In-house developed software (MATLAB ver. R2010a, Math Works Inc, Natick MA, USA) was used to calculate apparent diffusion coefficient (ADC) maps by fitting a mono-exponential model to the signal intensity of the images with different b-values, while T_2_-maps were calculated by fitting a mono-exponential model to the signal intensity of the images with different TE-values. The same software was also used to calculate DCE parameters using the average signal intensities within the region of interest specified below. The following DCE parameters were calculated based on the T_1_-weighted image series acquired during and after gadolinium-based contrast injection: relative signal intensity (RSI) 1.5 min after contrast injection, *RSI*_*1.5min*_, area under the curve (AUC) during the five first min after contrast injection, *AUC*_*5min*_ and time to peak signal (TTP).

**Fig. 1:**
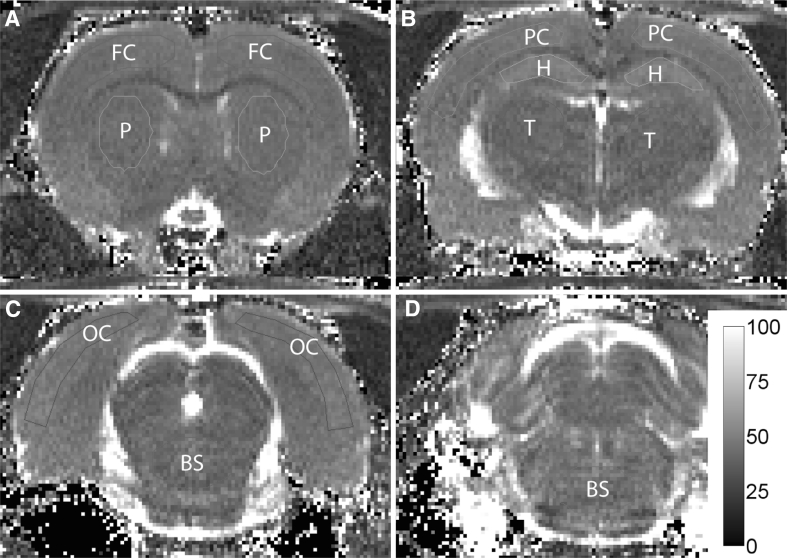
T_2_-maps of image slices throughout the brain showing regions of interest used in the analyses. *FC* frontal cortex, *P* Putamen, *PC* Parietal cortex, *H* Hippocampus, *T* Thalamus, *OC* occipital cortex, *BS* brain stem. *Colour bar* indicates T_2_-values (ms)

All MR images were visually evaluated with respect to morphological changes and with abnormal signal areas. In addition, Medical Image Processing, Analysis and Visualization software (ver. 5.3.4, Centre for Information Technology (CIT), National Institutes of Health (NIH)) (McAuliffe et al. 2001) were used for regions of interest (ROI) analyses of the image data. ROIs in the frontal, parietal and occipital cortex, hippocampus, putamen, thalamus and brain stem at the level of pons were drawn in the T_2_-maps (Fig. 1) and copied to the ADC-maps and T_1_-weighted images to ensure the same placement of ROIs in all image sets. Average voxel values in each of these regions were calculated and compared between groups. The same ROIs were also used to calculate DCE parameters, as described above. For the T_1_-weighted images used for manganese-enhanced MRI 1 week after the dive, ROI were also drawn in muscle areas lateral to the brain on both sides. Using the average signal intensity from muscle, the mean relative contrast (RC) was calculated in all the other ROI using the formula:

$$ \overline{RC}_{ROI} \; = \;\frac{{\overline{SI}_{ROI} }}{{\overline{SI}_{muscle} }} $$

Where *RC*_*ROI*_ is the relative contrast and *SI*_*ROI*_ is the mean signal intensity of the region of interest and *SI*_*muscle*_ is the mean signal intensity from the muscle ROI.

DTI analyses were performed with the tools of the FMRIB software library (FSL ver. 4.1.4, Oxford Centre for Functional MRI of the Brain, UK; www.fmrib.ox.ac.uk/fsl). Images were pre-processed to reduce image artefacts due to motion and eddy current distortions by affine transformation and co-registration of the diffusion encoded images to the *b*_0_ images. Single data sets with severe ghosting artefacts were excluded from further analyses (day 21: *n* = 6, day 42: *n* = 5). Brains segmented out using the Brain Extraction Tool before FDT ver2.0 (both part of FSL) was used to fit a voxel wise diffusion tensor model to the diffusion image data (Behrens et al. 2003). Maps for the fractional anisotropy (FA), mean (MD), radial (RD) and axial diffusivity were created for all animals for days 7 and 14 after decompression. ROI were drawn in the internal capsule, external capsule, hippocampal fimbria, body and splenium of corpus callosum on the FA-maps. Mean FA, MD, RD and axial diffusivity were calculated in each ROI in each animal.

### Histology

Two weeks after decompression, the rats were sacrificed with an overdose of pentobarbital (300 mg/kg) and perfused with 4 % paraformaldehyde in phosphate-buffered saline. Brains were post-fixed in the same fixative and embedded in paraffin, then cut in 8 μm thick coronal slices corresponding to −3.25 and −10.30 mm from the bregma (Paxinos and Watson 2008) and stained with hematoxylin–eosin (H&E) (Cell Path Ltd, UK, Chemiteknikk, Norway and Sigma Aldrich, Germany). The sections were also stained with Luxol fast blue (Chemiteknikk, Norway) for myelin. For immunohistochemistry, the paraffin sections were incubated with antibodies against anti-MAP-2 for neuronal integrity (Sigma, USA), anti-glial fibrillary protein (anti-GFAP) (Millipore, Norway) as a marker for reactive astrocytes, anti-cleaved caspase 3 (Cell Signaling Technology Inc., USA) for apoptotic calls, anti-myelin basic protein (anti-MBP) (Covance, USA) for myelin, anti-CD68:FITC (AbD Serotec, Germany) for activated microglia/macrophages and rabbit anti-rat albumin (Nordic, the Netherlands) for BBB leakage. After primary antibody incubation, sections were incubated with rat-anti-FITC-biotin (Roche, Basel, Switzerland), horse-anti-mouse-biotin (Vector Laboratories, Burlingame, CA), goat- anti-rabbit (Vector Laboratories, USA) or Dako Envision+System-HRP (Dako, Denmark). Visualization was performed using a Vectastain ABC kit (Vector Laboratories Inc., USA) and Diaminobenzidine (DAB) kit (Vector Laboratories Inc., USA).

The sections were examined for neuropathology using a Nikon Eclipse 80i light microscope and were analysed by a blinded investigator. To assess any gliosis or vascular proliferation both hemispheres were examined, and the number of GFAP-reactive astrocytes and albumin-labelled vessels were calculated using a 40× objective with an ocular grid.

### Statistics

PASW Statistics 18 (release 18.0.2, SPSS Inc., Chicago, IL, USA) was used for all statistical analysis and the level of significance was set to 0.05. Two-sided *t* tests were used to analyze differences in ADC, T_2_, DCE parameters, relative contrast in the T_1_-weighted images, and DTI parameters between groups of decompressed and control animals.

Histology was analysed by a semi quantitative method by a blinded and trained neuropathologist (SHT).

## Results

### Bubble formation and clinical outcome

Of the nine rats in the diving group, three died and six had bubble loads graded between 2 and 4 (median 2) following decompression. The rats that died had considerable bubble loads (visible in tissue after sacrifice), with two dying immediately after the dive and the third showing neurological symptoms of DCS in the form of temporary paralysis of the hind legs; it died after the first MRI scan. The remaining six animals had no clinical symptoms.

### Acute MRI findings

**Fig. 2:**
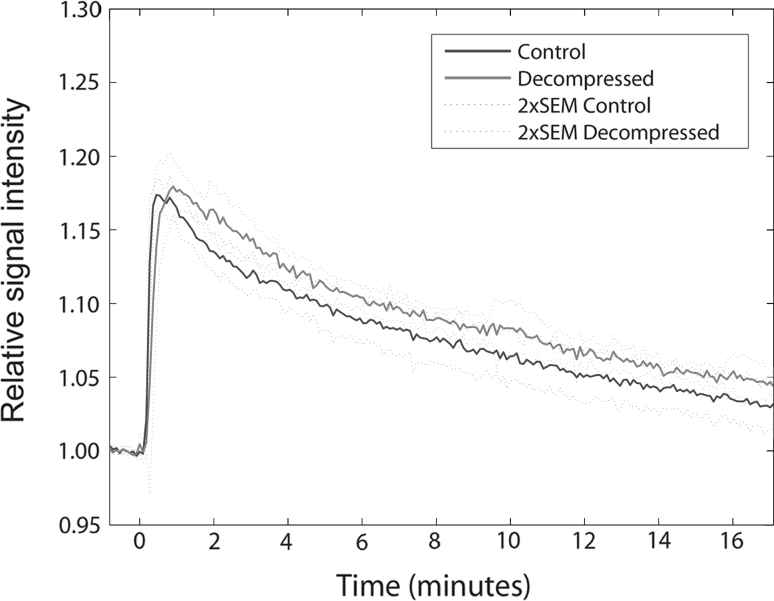
*Plot* of the mean relative signal intensity for the whole brain (cerebrum) with time after injection of contrast agent. *Blue line* represent mean for control animals with 95 % CI (*blue dotted line*) and *red line* represent mean for decompressed animals with 95 % CI (*red dotted line*)

Decompressed rats had a different time-curve for the dynamic contrast-enhanced MR signal than controls, with higher RSI (*P* = 0.017), a tendency towards longer time to peak (*P* = 0.11) and a larger area under the curve for the whole brain (*P* = 0.099) (Fig. 2). Region of interest based analyses of the dynamic contrast-enhanced MRI (DCE MRI) data that showed differences between decompressed and control animals in frontal cortex and thalamus. Decompressed animals had higher mean RSI (*P* = 0.023, Table 1) and a tendency towards higher TTP and AUC in the frontal cortex than control animals (*P* = 0.076 and *P* = 0.068, respectively). In the thalamus, AUC was higher among decompressed animals (*P* = 0.033, Table 1) with a tendency towards higher RSI and TTP (*P* = 0.068 and *P* = 0.104, respectively). For all other areas, there were no significant differences between the two groups.

**Table 1:** Calculated parameters from the dynamic contrast-enhanced imaging

	RSI_1.5min_				TTP (min)				AUC_5min_			
	Control		Decomp.		Control		Decomp.		Control		Decomp.	
	Mean	SD	Mean	SD	Mean	SD	Mean	SD	Mean	SD	Mean	SD
Frontal cortex	1.05	0.02	1.09*	0.02	0.20	0.05	0.88^+^	0.64	0.17	0.08	0.31^+^	0.11
Parietal cortex	1.10	0.03	1.09	0.02	0.36	0.07	0.67	0.37	0.40	0.19	0.34	0.07
Occipital cortex	1.09	0.03	1.11	0.01	0.56	0.19	0.81	0.59	0.38	0.12	0.41	0.07
Hippocampus	1.09	0.03	1.11	0.02	0.65	0.36	1.06	0.46	0.37	0.14	0.47	0.06
Putamen	1.08	0.01	1.09	0.02	0.54	0.26	1.17^+^	0.59	0.35	0.07	0.38	0.03
Thalamus	1.08	0.01	1.10^+^	0.02	0.43	0.28	1.62	1.27	0.35	0.03	0.44*	0.06
Brain stem	1.07	0.03	1.10	0.04	0.97	0.63	2.27	1.54	0.33	0.05	0.35	0.11

Mean relative signal intensity 1.5 min after injection (RSI_1.5min_), time to peak after injection (TTP) in min and area under the curve 5 min from injection (AUC_5min_) with standard deviation (SD) in different brain areas in groups of control and decompressed rats acutely after decompression

*P* values from two-sided *t* test with unequal variances

Significance is indicated by * *P* < 0.05, ** *P* < 0.01

*P* < 0.1 is indicated by^+^

Interestingly, the variation in all three parameters (RSI, TTP and AUC) was higher among decompressed animals than controls. There were no specific areas with increased signal in the post-contrast image compared to the pre-contrast image. The ADC-maps and T_2_-maps did not show any focal changes indicating pathology in neither the decompressed nor the control animals. Measurements of ADC and T_2_ in several brain areas showed no differences between the groups (Tables 2 and 3).

**Table 2:** Apparent diffusion coefficient (ADC) in different brain areas acutely after decompression

	Control		Decomp.	
	Mean	SD	Mean	SD
Frontal cortex	776.3	54.5	795.4	67.1
Parietal cortex	778.8	80.5	817.9	65.4
Occipital cortex	826.3	71.5	824.6	97.2
Hippocampus	830.0	56.4	879.3	74.4
Putamen	761.6	38.9	765.4	40.3
Thalamus	783.3	47.9	814.2	65.4
Brain stem	856.3	80.6	849.0	117.8

Mean ADC (10^−6^ mm^2^/s) with standard deviation (SD) in groups of control and decompressed rats

**Table 3:** T_2_ in brain areas at different times after decompression

	Acute			1 week					2 weeks			
	Control		Decomp.	Control			Decomp.		Control		Decomp.	
	Mean	SD	Mean	SD	Mean	SD	Mean	SD	Mean	SD	Mean	SD
Frontal cortex	49.5	1.1	50.1	1.5	47.1	0.6	47.5	0.9	48.1	0.6	48.3	0.9
Parietal cortex	50.8	0.6	50.9	1.5	48.4	0.5	49.1*	0.5	49.8	0.6	50.0	0.8
Occipital cortex	52.9	0.9	52.3	1.8	50.4	1.2	51.0	0.5	52.0	0.6	52.2	0.5
Hippocampus	55.1	0.3	55.1	1.4	51.3	0.9	51.6	1.4	53.2	0.8	52.8	1.8
Putamen	49.2	1.4	50.5	1.4	45.1	1.3	45.4	1.5	46.6	0.6	47.2	1.0
Thalamus	48.0	1.0	48.4	1.3	43.4	0.6	44.1	0.7	44.9	0.6	45.8*	0.8
Brain stem	51.7	0.9	51.7	1.9	46.6	1.0	48.1*	1.1	48.5	0.6	49.9**	0.4

Mean T2 (ms) with standard deviation (SD) in groups of control and decompressed rats. Acutely, 1 and 2 weeks after decompression

*P* values from two-sided *t* test with unequal variances

Significance is indicated by * P < 0.05, ** P < 0.01

### MRI 1 and 2 weeks after decompression

The T_2_-maps showed no signal abnormalities or morphological changes 1 or 2 weeks after the decompression. Region of interest based measurements of T_2_ showed higher T_2_ in the brain stem among decompressed animals than controls after 1 and 2 weeks, with the same tendency seen in thalamus (Table 3). There was also a similar reduction in T_2_ in both groups from time 0 to week 1, with a slight increase to week 2 (Table 3) which was related to the administration of manganese on day 1 after decompression. Visual inspection of T_1_-weighted images after 1 week showed similar manganese-enhancement in both groups, and region based analysis of the images did not show any differences between groups in relative contrast 1 week after administration of MnCl_2_ (Table 4).

**Table 4:** Manganese-enhanced MRI

	Control		Decomp.	
	Mean	SD	Mean	SD
Frontal cortex	1.82	0.37	2.05	0.16
Parietal cortex	1.91	0.29	2.04	0.13
Occipital cortex	1.75	0.12	1.73	0.11
Hippocampus	2.10	0.28	2.04	0.16
Putamen	1.97	0.22	1.95	0.15
Thalamus	1.93	0.18	1.82	0.18
Brain stem	1.90	0.16	1.82	0.13

Mean relative contrast with standard deviation (SD) on T_1_-weighted MRI in different brain areas in groups of control and decompressed rats after 1 week. Increased relative contrast indicated manganese-enhancement

**Fig. 3:**
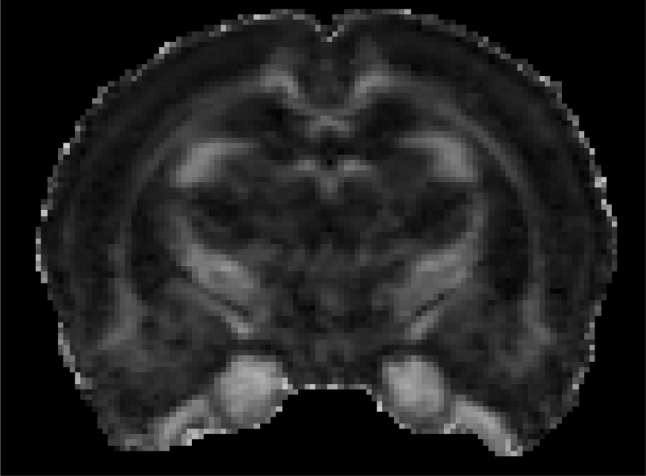
Image shows representative fractional anisotropy (*FA*) map

No specific white matter abnormalities were seen on DTI and parameters such as fractional anisotropy (FA) (Fig. 3), mean, axial and radial diffusivity were not different between groups in the measured white matter structures (Table 5).

**Table 5:** Diffusion Tensor Imaging results

	Week 1				Week 2			
	Control		Decomp.		Control		Decomp.	
	Mean	SD	Mean	SD	Mean	SD	Mean	SD
Corpus callosum body	0.76	0.03	0.73	0.08	0.73	0.04	0.75	0.03
Corpus callosum splenium	0.79	0.03	0.73	0.08	0.69	0.04	0.67	0.08
External capsule	0.48	0.01	0.47	0.03	0.46	0.01	0.47	0.02
Hippocampal fimbria	0.71	0.03	0.65	0.06	0.75	0.01	0.73	0.04
Internal capsule	0.67	0.05	0.63	0.06	0.73	0.06	0.75	0.02
All structures	0.58	0.02	0.55	0.03	0.58	0.02	0.58	0.01

Mean fractional anisotropy (FA) and standard deviation (SD) in different white matter structures in groups of control and decompressed rats after 1 week

*P* values from two-sided *t* test with unequal variances

### Histology

**Fig. 4:**
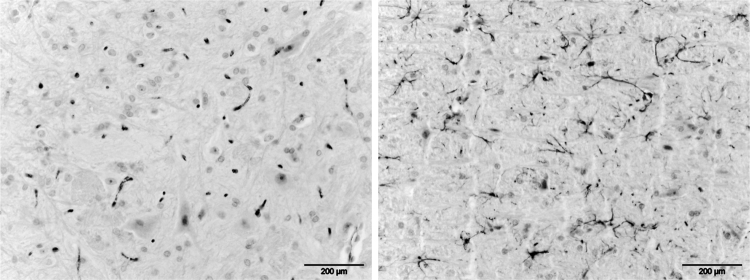
Albumin and GFAP stain from brain stem

In routine haematoxylin and eosin (H&E) sections and after histochemical and immunohistochemical analyses, no histopathological changes were observed with regard to neuropil, neurons, glial cells, vessels or leptomeninges. In particular, no signs of degeneration of neurons (no red neurons), demyelination, gliosis, inflammation, microglial activation or endothelial injury were detected. Furthermore, no parenchymal immunostaining for albumin was noted consistent with no vascular leakage (Fig. 4).

## Discussion

The present study is, to our knowledge, the first controlled, longitudinal study where MRI has been used to evaluate effects of decompression. The results indicate that decompression causes altered brain perfusion in the acute phase, with increased T_2_ in the brain stem on MRI in the 2 weeks after decompression.

To study the ongoing process of the physiological responses to a dive, three time points were chosen for measurement, at 1 h, 1 and 2 weeks after decompression in experimental animals. The dive profile that we used caused bubble formation in all rats, with neurological symptoms (paresis of the hind limbs) noted in one rat within 24 h and immediate death caused in two rats. The death of three rats indicates the severity of the dive profile. The rats that died had considerable amounts of gas present in their vascular system and tissue. Therefore, death was most probably due to a large number of vascular gas bubbles causing cardio-vascular collapse and shock leading to death (Muth and Shank 2000).

The first MRI measurements were designed to look for acute effects in the brain. DCE MRI was performed to assess brain perfusion and permeability of the BBB. The gadolinium-based contrast agent, Omniscan, used in this study remains in the vasculature under normal homeostasis, but is known to leak across the BBB when its integrity is compromised (Wardlaw et al. 2008). The signal change and shape of the signal curve reflects the amount of contrast agent in the brain circulation and any redistribution of the contrast agent from the circulation to the brain tissue. In the present study, the DCE measurements showed that decompressed rats had increased RSI and AUC compared to controls. This indicates an increased blood flow to the brain tissue, and may be explained by an increased oxygen demand secondary to a tissue hypoxia–ischemia caused by the decompression stress. Perfusion changes are previously documented in occupational divers in watershed areas of the brain on MRI, and are explained by possible changes in the function of the cerebral microvasculature (Moen et al. 2010). Since DCE was only performed at one time point in our study, the duration of such circulatory changes are uncertain.

As the shape of the signal curves from the DCE in the elimination phase was similar between decompressed and control animals and there were no areas with increased signal intensity in the post-contrast images compared to the pre-contrast images, it was concluded that there was no significant leakage over the BBB in response to the decompression at the time of imaging in the present study. Decompression has previously been shown to cause increased permeability of the BBB, as illustrated by leakage of dye (Trypan blue) into brain tissue of decompressed rabbits with dye concentration correlating to visible intravascular bubbles (Chryssanthou et al. 1977). We did, however, not find any leakage of albumin in our immunostained brain tissue sections, which would have indicated a BBB leakage. However, using histochemical methods such as Evans blue, increased BBB permeability after decompression has been shown to be temporary and reversible (Nohara and Yusa 1997). In the present study, the acute MRI acquisition started 1 h after the decompression and the DCE was performed last of all imaging, starting approximately 2 h after the decompression. Hence, any increased permeability of the BBB may have been reversed at the time of imaging. Future studies should, therefore, aim to perform DCE imaging immediately after the decompression to establish whether this dive protocol causes temporary changes to the BBB permeability. It is important to establish whether there is a change in BBB permeability in response to diving, since this may have consequences for drug administration to divers and compressed air workers. If the BBB is more permeable at a certain time point, then drugs and environmental toxins may penetrate the brain in amounts that could produce toxic or undesirable effects (Chryssanthou et al. 1977). Thus, changes in the integrity of the BBB do not necessarily have any adverse effects per se, but exposure to potentially damaging drugs or environments while it is compromised may do.

Except for the changes in DCE MRI, no focal or general changes indicating cerebral injury were found on anatomical (T_1_ and T_2_) or diffusion weighted imaging 1 h after the decompression. The early time point of imaging may explain lack of signal changes on T_1_ and T_2_, but diffusion weighted imaging has previously been shown to be a very sensitive method for detecting ischemic lesions (van Everdingen et al. 1998). However, the lack of signs of injury on acute MRI is not surprising, since all but one of the animals showed no neurological signs at that time. In previous studies, focal CNS injuries on conventional MRI were found in the presence of neurological symptoms and signs (Yoshiyama et al. 2007; Jallul et al. 2007; Gronning et al. 2005; Reuter et al. 1997). Moreover, even in the presence of clinical neurological signs suggesting brain involvement, abnormalities on cerebral MRI are uncommon, occurring only in 25 % of the patients in one study (Reuter et al. 1997) and none in another (Gronning et al. 2005).

Hence, lack of detectable injury on acute MRI does not exclude the presence of cerebral injury or that injury can evolve. In fact, on follow-up 1 and 2 weeks after the decompression, changes in T_2_ were found in brain stem and thalamus among decompressed animals. These delayed findings are in accordance with previously published clinical reports, where MRI abnormalities, especially increased T_2_ indicating focal CNS injuries, have been found several weeks after the decompression (Reuter et al. 1997; Hierholzer et al. 2000; Jallul et al. 2007). These studies showed MRI abnormalities mostly in the spinal cord, and this is despite the fact that MRIs made during the first 24 h after onset of symptoms (Jallul et al. 2007) were normal. In the present study, we did not image the spine, but the T_2_ changes were located in the basal parts of the brain. These brain areas are supplied by the basal artery and may have poorer collateral circulation than the neo-cortex, and thus represent more vulnerable areas of the brain in case of large arterial air embolism. The primary cause of increased T_2_ is increased tissue water content that can be taken as an indication of tissue injury with secondary edema. Reduced capillary density may also lead to increased T2 (Norris 2006). However, histological examinations did not show any signs of tissue injury or vascular changes in the areas with increased T_2_ or in any other brain areas. The origin of the increased T_2_ is, therefore, uncertain.

The lack of other findings on MRI after the acute phase could mean that the technique is not sensitive enough to detect subtle tissue changes. However, the MRI results were supported by histological and immunohistochemical examinations that did not show any structural changes in any part of the brain.

One of the hypotheses of this study was that decompression leads to cerebral white matter injuries. However, such injuries may be too subtle to detect on histological examination and anatomical MR imaging, but could be detected using more functional imaging techniques such as DTI where the quantity and directionality of the water diffusion of the tissue can be measured. Injuries to axons and reduced myelination both give specific changes in the quantity and directionality of water diffusion, but no such effects could be detected in our study. Hence, there were no indications that the decompression caused any harmful effects to central cerebral white matter.

There have been several other attempts to study long-term effects in animal models; the CNS has been studied in goats subjected to several (mean 12.5), relatively severe dives over a number of years. In the brains of three out of 36 animals, lesions were seen on MRI (T_2_). However, this was seen in goats with no history of DCS. No evidence of any histopathological damage in the brains of dived animals was found through staining with H&E and glial fibrillary acidic protein (GFAP) (Blogg et al. 2004; Woodger et al. 2001). One suggestion for the lack of significant brain lesions detected in these dived goats was that goats have a slightly different carotid blood supply than humans, with a rete that might act as a filter to some bubbles that pass into the arterial circulation (Daniel et al. 1953).

## Conclusions

In conclusion, the present study indicates that severe decompression does not seem to cause any structural or cellular injury to the brain tissue of the rat, but may cause circulatory changes in the brain perfusion.

## Abbreviations

ADC: Apparent diffusion coefficient
AUC: Area under the curve
BBB: Blood brain barrier
CNS: Central nervous system
DCE: Dynamic contrast enhanced
DCS: Decompression sickness
DTI: Diffusion tensor imaging
EEG: Electroencephalography
FA: Fractional anisotropy
FLASH: Fast low angle shot
FOV: Field of view
MD: Mean diffusivity
MRI: Magnetic resonance imaging
MTX: Acquisition matrix
RARE: Rapid acquisition with relaxation enhancement
RD: Radial diffusivity
RC: Relative contrast
ROI: Region(s) of interest
RSI: Relative signal intensity
SD: Standard deviation
T_1_: Longitudinal relaxation time
T_2_: Transverse relaxation time
TE: Echo time
TR: Repetition time
TTP: Time to peak
